# Big data in wildlife research: remote web-based monitoring of hibernating black bears

**DOI:** 10.1186/s12899-014-0013-1

**Published:** 2014-12-11

**Authors:** Timothy G Laske, David L Garshelis, Paul A Iaizzo

**Affiliations:** Department of Surgery, University of Minnesota, B172 Mayo, MMC 195 420 Delaware Street SE, Minneapolis, MN 55455 USA; Cardiac Rhythm and Heart Failure, Medtronic, Incorporated, 8200 Coral Sea Street NE, MVS46, Mounds View, MN 55112 USA; Minnesota Department of Natural Resources, 1201 E Hwy 2, Grand Rapids, MN 55744 USA

**Keywords:** American black bear, Hibernation physiology, Heart rate, Implantable cardiac monitor, Wireless data transmission

## Abstract

**Background:**

Numerous innovations for the management and collection of “big data” have arisen in the field of medicine, including implantable computers and sensors, wireless data transmission, and web-based repositories for collecting and organizing information. Recently, human clinical devices have been deployed in captive and free-ranging wildlife to aid in the characterization of both normal physiology and the interaction of animals with their environment, including reactions to humans. Although these devices have had a significant impact on the types and quantities of information that can be collected, their utility has been limited by internal memory capacities, the efforts required to extract and analyze information, and by the necessity to handle the animals in order to retrieve stored data.

**Results:**

We surgically implanted miniaturized cardiac monitors (1.2 cc, Reveal LINQ™, Medtronic Inc.), a newly developed human clinical system, into hibernating wild American black bears (N = 6). These devices include wireless capabilities, which enabled frequent transmissions of detailed physiological data from bears in their remote den sites to a web-based data storage and management system. Solar and battery powered telemetry stations transmitted detailed physiological data over the cellular network during the winter months. The system provided the transfer of large quantities of data in near-real time. Observations included changes in heart rhythms associated with birthing and caring for cubs, and in all bears, long periods without heart beats (up to 16 seconds) occurred during each respiratory cycle.

**Conclusions:**

For the first time, detailed physiological data were successfully transferred from an animal in the wild to a web-based data collection and management system, overcoming previous limitations on the quantities of data that could be transferred. The system provides an opportunity to detect unusual events as they are occurring, enabling investigation of the animal and site shortly afterwards. Although the current study was limited to bears in winter dens, we anticipate that future systems will transmit data from implantable monitors to wearable transmitters, allowing for big data transfer on non-stationary animals.

## Background

Wildlife research has long benefited from the use of behavioral and physiological monitoring devices [1–12]. Such devices have had significant impacts on both the types and quantities of information that can be collected, but their utilities have been limited by internal memory capacities, the efforts required to extract and analyze information, and by the requirement that the animals need to be handled in order to retrieve stored data. In parallel, innovations in the management and collection of “big data” have occurred in the field of medicine, resulting in substantial improvements in access to detailed clinical data in order to improve patient care [13–15]. Although human clinical devices have been successfully deployed in captive and free-ranging wildlife [16–18], emerging technologies with improved data recording and management capabilities hold the potential to provide even further insights into both normal physiology and the impacts of human interactions on animal behavior.

The Reveal LINQ™ Insertable Cardiac Monitor (ICM) is an implantable monitoring system that records subcutaneous electrocardiograms (ECGs) and is indicated for human clinical use for: 1) patients with clinical syndromes or situations at increased risk of cardiac arrhythmias and 2) patients who experience transient symptoms that may suggest a cardiac arrhythmia [19]. A common use of the system is for unexplained syncope (fainting), in which case the implanted device can capture episodes with impaired cardiac outputs, including bradycardias (unusually low heart rates), asystoles (long periods without a heart beat), or tachycardias (unusually high heart rates). For applications in wildlife research, the ability to capture extremes in heart rates may signal distinct physiological states, or ecological stressors or disturbances. These systems also could be used to record long term trends in heart rates, heart rate variability, and/or activity, enabling additional insights for wildlife monitoring.

Previous generations of this ICM have been deployed in captive brown bears (*Ursus arctos*) and wild American black bears (*Ursus americanus*) [16–18]. Although these devices provided substantial insights into both behaviors and physiologies, their capabilities were hampered by limitations in internal memory capacities. Specifically, electrocardiographic recordings in previous devices were limited to less than 30 minutes and 30 episodes. Once reaching that limit, older episodes were over-written by new ones. This is appropriate for human clinical applications since this typically allows for the diagnoses of the arrhythmias of interest. In addition, following an event, the human patient can seek medical attention at which time the device memory can be queried. By contrast, the device memories are likely to fill up in free-ranging wildlife, which cannot be regularly handled. In this study we sought to overcome these limitations by downloading data at 2-hour intervals to a web-based data storage and management system, thereby dramatically increasing the recording resolution.

Our study subjects were hibernating black bears at their natural den sites in the wild. In most of their ranges, black bears spend 4–6 months of the year in a state of dormancy, with minimal physical activities, without food or water, in a state of mild hypothermia (~30-36°C), and typically without urinating or defecating [11]. Hibernating females give birth during mid-winter and also den with these cubs in the subsequent year [20]. It should be noted that some bears spend the winter in either partially-exposed dens or open nests with associated higher risks for predation and/or external disturbance [12,21]. Importantly, bears can defend themselves and their offspring through their maintenance of muscle strength and their capability of rousing from hibernation within seconds [22–24]. Black bears commonly elicit defensive posturing and high respiratory rates within seconds of being disturbed, with their hearts transitioning from the quiescent state of hibernation, to supporting bursts of activity [18].

Results of earlier studies indicate that cardiac wall thickness and function (electrophysiological parameters) were maintained in bears during the period of hibernation [17]. A respiratory sinus arrhythmia (RSA) enables the heart to rest between inspirations, with cardiac pauses of up to 14.4 seconds previously documented [18]. This RSA is thought to be adaptive to conserve energy while maintaining adequate cardiac function to sustain their “fight or flight” responses [18]. Although substantial research has been performed on ursid hearts during hibernation, limitations have included data collection at discrete time intervals, limited device memories, high foreign body rejection rates, and data collection from animals that were anesthetized, disturbed, or captive [16–18,25–28]. A primary goal of this study was to further characterize the unique physiology of these animals by removing the previous limitations associated with the volume of data that could be collected, as well as to provide near-real time information about the state of the hibernating bear. In addition to improved management of wild and captive populations, a more detailed understanding of hibernation physiology may provide new insights into therapeutic approaches in human medicine [29–31].

## Methods

**Figure 1:**
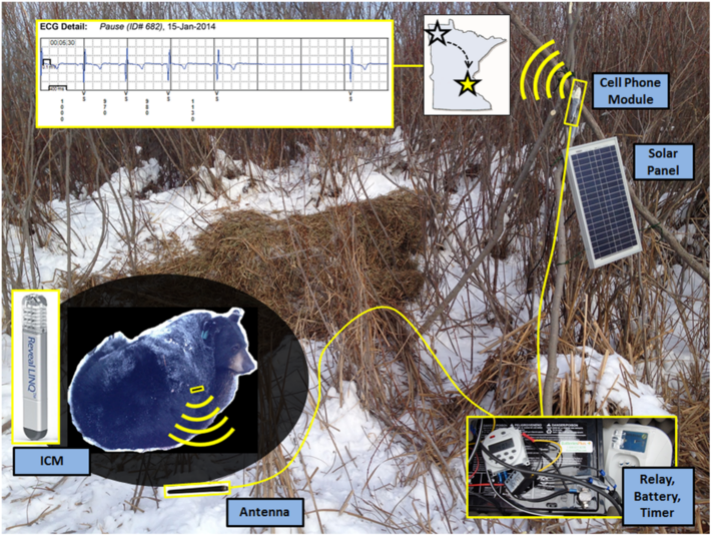
**Wireless telemetry system at bear den.** The insertable cardiac monitor (ICM) communicated with a relay station housed in a waterproof container via an antenna buried under the bear. Transmissions to an internet site were via a cellular module attached to a timber tripod fabricated at the site. The system was powered by 12 volt batteries charged by a solar panel.

**Figure 2:**
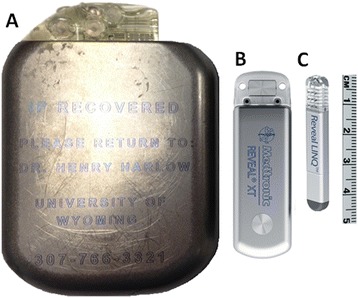
**Three generations of implantable monitors used by this research team.** Device **A** has a volume of 80 cc [17], device **B** a volume of 9 cc [18], and device **C** (used in this current study) a volume of 1.2 cc. A millimeter ruler is included as a size reference.

Free-ranging, wild radiocollared bears in northern Minnesota (46.0 – 48.5° N latitude; N = 6) were located in their winter dens in mid-December, anesthetized (Telazol®, veterinary formulation of tiletamine and zolazepam, Elkins-Sinn, Inc., Cherry Hill, NJ, 4.4 mg/kg) and temporarily extricated. Ethylene Oxide sterilized Insertable Cardiac Monitors (ICMs) that were developed for human heart patients (Reveal LINQ™, Medtronic Inc., Minneapolis, MN; 1.2 cc; 4.0 mm × 7.2 mm × 44.8 mm; 2.4 grams), were surgically implanted in these bears in the field using aseptic techniques (Figures 1 and 2). Devices were placed subcutaneously in left peristernal locations with surgical sutures used to close the puncture sites. Device programming was performed using transcutaneous telemetry (CareLink® Model 2090 Programmer with software Model SW026, Medtronic Inc., Minneapolis, MN) and mid-winter data retrieval utilized wireless telemetry from a relay station (Medtronic MyCareLink® Patient Monitor Model 24950, Medtronic Inc., Minneapolis, MN).

The ICM records the average daytime heart rates (HRs) for the period from 08:00–20:00 (referencing a 24 hour clock) and average nighttime HRs for the period of 0:00–04:00 through analyses of the subcutaneous electrocardiogram recorded by the devices. The total daily activities (minutes/day) were derived from an accelerometer housed within each device. Arrhythmias that can be selected for automatic detection, including a detailed ECG tracing, include: atrial tachyarrhythmias/atrial fibrillation (AT/AF), bradyarrhythmias, asystoles, and ventricular tachyarrhythmias. In this study, the devices were programmed at the den sites to automatically detect and store ECGs for episodes in which: 1) a heart rate of at least 176 beats per minutes (bpm) was sustained for at least 16 consecutive beats (“ventricular tachycardia”) and 2) if a pause of at least 4.5 seconds occurred between consecutive heart beats (“asystole”). A 10-second sample of the current ECG is also included during data transmissions.

**Figure 3:**
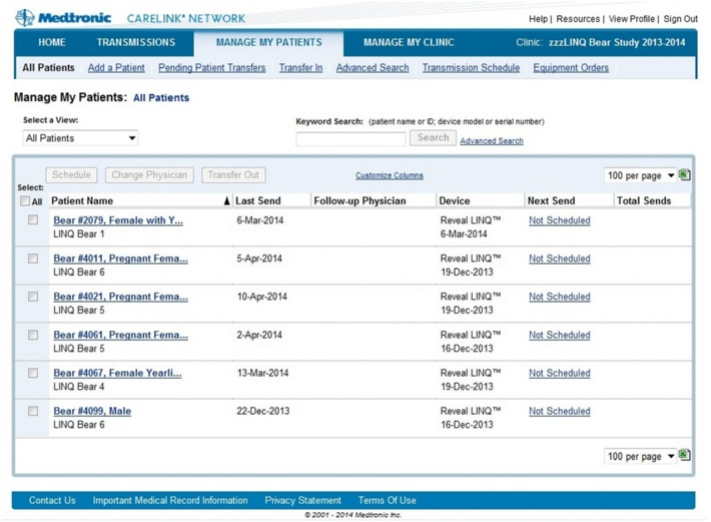
**Image of website used for data tracking and display.** The subject bears were enrolled into a web-based clinic as patients. The site served as both a repository for data and as a means of tracking transmissions. In addition, the site automatically alerted the user when an event of interest occurred.

To enable collection of continuous, near-real time mid-winter data, remote telemetry controls and power systems were constructed and deployed near each den site (Figure 1). An antenna was buried ~10 cm below the floor of each underground den or aboveground nest (within the soil or nesting material) to allow reliable wireless communication to the device implanted in the bear. A 5-meter long coaxial cable connected the antenna to the relay station, which was housed within an insulated, water-tight box positioned near the den. This box also housed batteries (either two 55 amp hour batteries: Model WKDC12-55P or a single 100 amp hour battery: Model WKDC12-100P; Werker), a DC converter (12 V to 5 V 15 W DC/DC converter Model C120503, CPT), a digital timer (12 V digital timer Model CN101A, Amico), and a solar panel controller (20 W Model ASC20, EcoEnergy, Canada). A tripod was constructed from saplings at each site to allow positioning of a solar panel (20WModel Number SLP020-12U, Solarland, Ontario, CA) and the cell phone module, which was directly wired to the patient monitor (elevated ~2 meters above the ground using a USB extension). Each system was duty-cycled using the digital timer (powered on for 20 minutes every 2 hours) to ensure adequate power availability over the 3-month study period. The devices were programmed to transmit heart rate and activity data every 2 hours to a website housed in Minneapolis, Minnesota, established for managing human clinical data (Figure 3). Average ventricular heart rates, heart rate variability, and activity were automatically plotted and stored on the website. The website also immediately alerted us to significant changes to any of these parameters.

In December 2013, we implanted cardiac monitors in 6 hibernating black bears (3 pregnant females, 2 females with yearlings, 1 adult male). Follow-up visits were made to each den site during early March, 2014 to check on the health of the bears and to examine the integrity of each telemetry system. During the study period (mid-December 2013 to mid-April 2014) the ambient environmental temperature ranged from −38.9 to 16.1°C.

All studies were conducted in conjunction with the Minnesota Department of Natural Resources and were also approved by the University of Minnesota’s Animal Care and Use Committee.

## Results and discussion

Data transmissions were received from 4 of the 6 systems over the winter months (Table 1). Telemetered data were not received from the adult male, because he left his aboveground nest, or from one female with yearlings, because the bears had chewed through the antenna within the den. The acquisition of data increased from a maximum of 30 episodes with ECGs in prior generations of the ICM to up to 137 episodes with ECGs plus 517 additional sample ECGs (associated with transmissions) using the new technology.

**Table 1:** **Summary information for the bears that were successfully monitored**

**Bear ID**	**Implant date**	**Follow-up visit date**	**Date and time of last transmission**	Total number of asystolic events*	**Number of episodes with ECGs transmitted**	**Description**
4061	19-Dec-2013	11-Mar-2014	02-Apr-2014 08:35	15,211	85 asystoles (ranging from 4.5 to 5 seconds)	Pregnant female. 3 female cubs born.
				(No events for 8 days: 03-Jan to 10-Jan)		
4021	19-Dec-2013	09-Mar-2014	10-Apr-2014 00:05	12,773	69 asystoles (ranging from 4.5 to 6 seconds)	Pregnant female. 2 female cubs born.
				(No events for 23 days: 23-Dec to 14-Jan)		
4067	20-Dec-2013	10-Mar-2014	13-Mar-2014 06:05	65,535 as of 01-Feb-2014**	103 asystoles (ranging from 4.5 to 16 seconds); 2 tachycardias (207 and 222 beats per minute)	Female denning with 2 yearlings.
4011	21-Dec-2013	10-Mar-2014	05-Apr-2014 10:05	2,089	137 asystoles (ranging from 4.5 to 5 seconds)	Pregnant female. 3 female cubs born.
				(No events for 18 days: 24-Dec to 09-Jan)		

*Asystolic events are defined as pauses between consecutive heartbeats of at least 4.5 seconds.

**Note that there was 16 bits allocated to the counter and hence 65,535 was the greatest value that could be recorded. The extreme number of events recorded in the bears was beyond what was anticipated in human clinical use. Even though the counter was limited, the device continued to collect and transmit data. This number of events was reached on 01-Feb-2014. Bear #4067 averaged 1584 events/day (1.1 asystolic events/minute) during January 2014 prior to the limit being reached.

**Figure 4:**
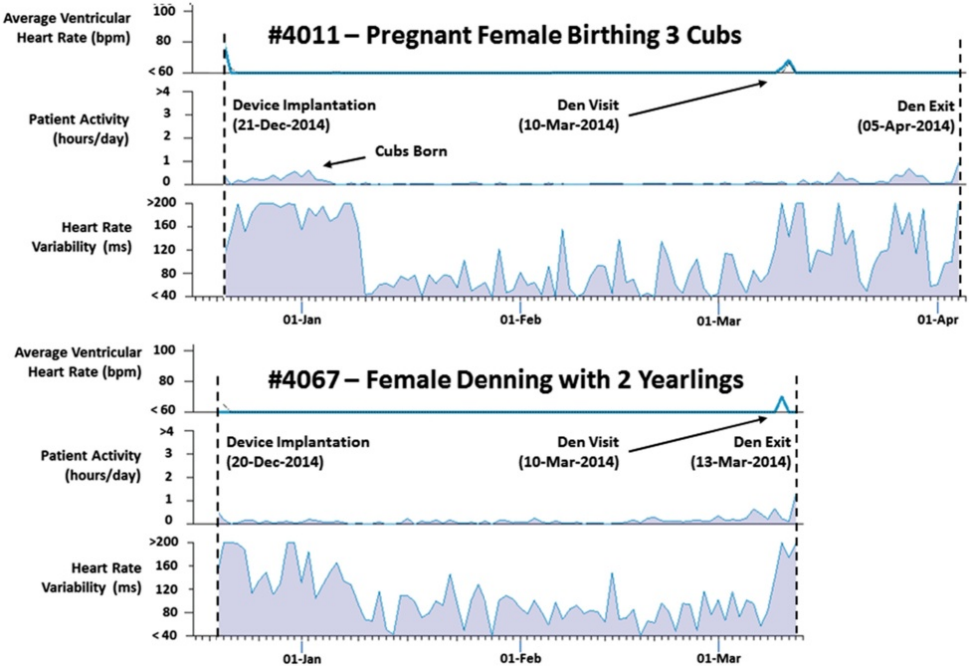
**Example near-real time data acquired from denning bears.** A dramatic reduction in activity and heart rate variability was observed in pregnant females post parturition (such as 4011 shown here), whereas a constant level of activity and heart rate variability was maintained throughout the winter for the female denning with yearlings (4067). (Note: To calculate heart rate variability, the device measures each ventricular interval and calculates the median interval every 5 min. The heart rate variability value (in ms) for each day is then calculated as the variability seen in the 5-minute median values over the 24-hour period [19]).

**Figure 5:**
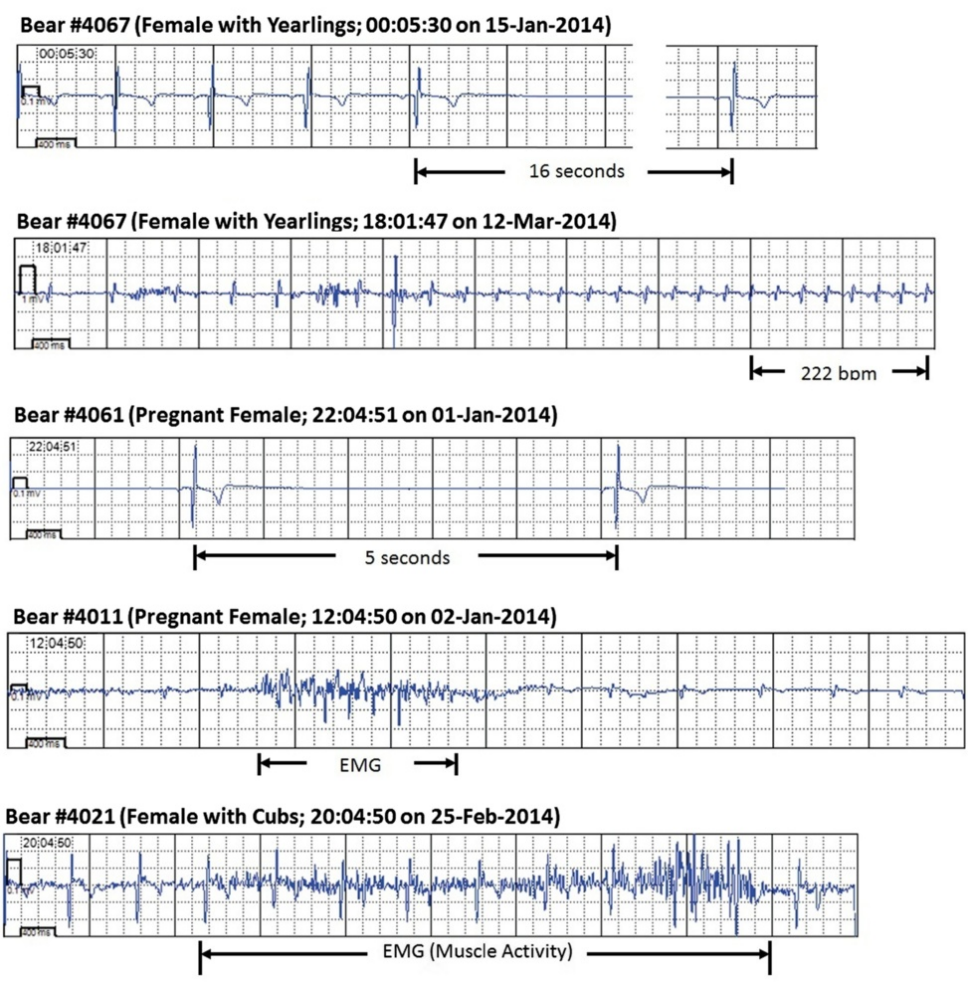
**Sample ECG plots acquired from denning bears.** The top two panels show the minimum (16-second pause) and maximum heart rates (222 beats/minute) recorded during the study period. Both were recorded from bear 4067. The maximum rate occurred the day following our visit to the den and is believed to be associated with a subsequent visit to the den by the landowners. A 5-second pause is shown for bear 4061 in the third recording and the bottom two recordings include myograms relating to skeletal muscle activity (during the period of birthing for 4011 and post-parturition for 4021).

Heart rate patterns exhibiting an extreme respiratory sinus arrhythmia were elicited by all animals with regular sinus pauses (asystoles). In the pregnant females, increased movements, heart rates, and muscle activities and reductions in their RSAs occurred prior to parturition. Following birthing, near complete cessation of activities occurred and the magnitudes of the RSAs returned to levels consistent with that of the early winter. We suggest that the mothers remained more stationary immediately after birthing so as not to crush the altricial cubs, which stay warm beneath her for constant access to milk. Interestingly, the study female with yearlings did not elicit these patterns in heart rates and activity. In addition, from the females that birthed cubs, we recorded no sinus pause exceeding 6 seconds during the winter, whereas the female over-wintering with yearlings elicited consistently longer pauses (16 second maximum). During the period surrounding birthing, no sinus pause reached the minimum device recording threshold of 4.5 seconds for periods of 18, 23, and 8 days (bears 4011, 4021, and 4061, respectively; Table 1). Examples of heart rate and activity trends and electrocardiographic tracings for various states are shown in Figures 4 and 5.

**Figure 6:**
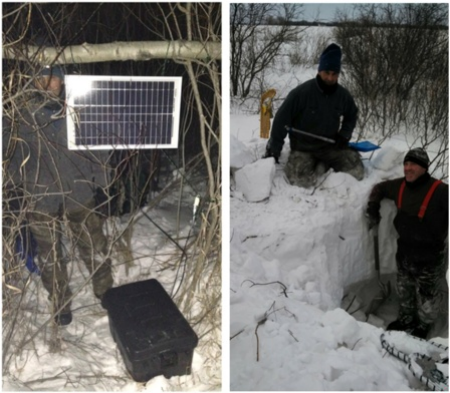
**Telemetry system covered by snow drift.** The bear at this site, which was denned in an open nest, relocated after our December visit. The site was exposed to heavy winds, causing the telemetry box and solar panel (left panel) to be covered by 2 m of snow in early March (right panel). Author Iaizzo appears in the left side of both photos and author Laske is standing on the black telemetry box in the right panel.

An important finding we report here was that the only significant disturbances to these hibernating bears were our visits to their dens, and in one case this was followed by a visit by the landowner (Figure 5). It should be noted that the male bear that vacated its open nest den at the edge of a field following our visit in December fortuitously escaped being covered under a snow drift (Figure 6). Importantly, these deployed remote monitoring systems allow for the immediate detection of unusual events such as a den disturbance or something physiologically abnormal with the bear, thus providing an opportunity to quickly check the den to observe what had occurred. For example, we previously observed denning bears that had survived an attack by wolves (*Canis lupus*), but our observations occurred months after the attacks, thus hindering our ability to reconstruct details of the events.

During our follow-up den visits in March, we found all bears to be in good condition, but 1 of the 6 ICMs had been rejected from under the skin via a foreign body response. We have seen this frequently in black bears [32,33], and had anticipated that the much smaller size of these newer devices would have eliminated the problem of rejection. We do not know when this occurred because the bears had already damaged the antenna inside this den, and we could not find the device inside the den with a metal detector. We implanted a second ICM in this female in March, but again the recording system failed. When we retrieved the system in the spring, we found that the antenna had again been damaged by the bears. Although data were not transmitted by this device, heart rate and activity trends will be downloaded using transcutaneous telemetry at a future date when handling the animal (assuming the second device remains implanted).

The number of data transmissions over the study period ranged from 125 (bear 4061) to 517 (bear 4011). Factors impacting the reliability of transmissions likely included battery temperature, exposure of the solar panel to sunlight, reliability of cellular phone coverage, and the position of the bear relative to the antenna. The impact of these factors will be investigated in future studies to enable further system refinement.

## Conclusions

This study is the first to wirelessly transmit detailed data on heart rhythms from animals in the wild. In addition, it uniquely demonstrated the ability to use a web-based data storage and management systems for recording and plotting cardiac events and trends associated with the physiological state and activity of wild bears. Further, the use of these devices allowed for continuous monitoring of heart rhythms and physical activities, with regular downloads of ECGs strips associated with notable cardiac events. These devices provided substantially more information than had been previously recorded, including detailed responses to birthing and cub-rearing during hibernation. They also provided an opportunity to observe physiological changes in near-real time. Although the current study was limited to bears in winter dens, we anticipate that future systems will transmit data from implantable monitors to wearable transmitters (e.g., radio-telemetry collars) and then to cell phone towers or satellites, just as today GPS data are transmitted. Such systems will allow for “big data” transfer from free-ranging animals to better understand physiologic responses and behaviors associated with factors such as climate change and interactions with humans.

### Consent

Written informed consent was obtained for the publication of this report and any accompanying images.
